# The Giving of Death Certificates

**Published:** 1899-10

**Authors:** 


### THE GIVING OF DEATH CERTIFICATES.

THE recent case of the death of the Gillespie child, on Milsom street, has more than ever drawn their attention to the slip-shod method of passing out death certificates in the coroner's office. Briefly, the facts are these: The child was eight months old, and died in the early morning hours without medical attendance. The parents had sent for a neighbor, a reported christian scientist, a Mrs. Hager, who is not, however, named as a healer in the official list of Buffalo scientists. After the child died a near-by physician, who had been sent for, arrived and gave a death certificate, naming convulsions as the cause.

This curiosity was passed by the coroner's office, but rejected by the Health Department, as insufficient and incomplete. Then Coroner Kenney, at the request of Health Commissioner Wende, ordered an autopsy, which was performed. There was found evidences of beginning congestion of the lungs, there was injection of the cerebral vessels, and the intestines were filled with feces.

The post mortem examiner made his report to the coroner, but the latter declined to make any investigation. Dr. Wende, on the contrary, sent out and made his own investigation in his own way and whether or not he achieves results matters little in this particular case; the fact remains that he is doing his duty as a public official, which is in strong contrast to the laxity or indifference at first manifested by the coroner.

The alleged christian science phase of this case is not its worst feature by any means. While we are fighting the great army of fakirs who practice medicine as christian scientists, faith curists, vodoo doctors, leg-pullers, clairvoyants, charm-workers, bone-throwers, snake-skinners and what-nots, we should not lose sight of the fact that these charlatans would not be able to cover up their malpractice, were it not that some regularly licensed and registered physician called after death usually gives a certificate.

The stamping out of these practices would be a comparatively easy matter, were it made obligatory on physicians to report to the Health Department all cases on which they had not been in attendance at least twenty-four hours before death.

The JOURNAL suggests that the report be made to the Health Department, because that office can be trusted to protect the community against evils that are threatening.

